# Supplementary material for: A Comprehensive Framework to Evaluate the Effects of Anterior Cruciate Ligament Injury and Reconstruction on Graft and Cartilage Status through the Analysis of MRI T2 Relaxation Time and Knee Laxity: A Pilot Study
Source: Life (Basel). 2021 Dec 10;11(12):1383. doi: 10.3390/life11121383 (PMC8706566; doi:10.3390/life11121383)
Supplement: Supplementary file 1 [file life-11-01383-s001.zip › life-1434063-supplementary/Correlations.pdf]

life

MDPI

Supplementary Materials: A Comprehensive Framework to Evaluate the Effects of Anterior Cruciate Ligament Injury and Reconstruction on Graft and Cartilage Status through the Analysis of MRI T2 Relaxation Time and Knee Laxity: A Pilot Study

C1. Relationship between cartilage and graft T2 values.

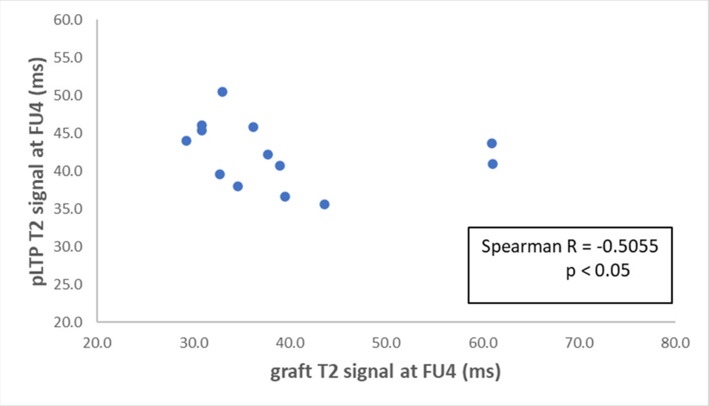

Figure S1. Distribution of the pLTP T2 signal (ms) at FU4 as a function of the graft T2 signal (ms) at FU4. Spearman's correlation details (p-value and R-coefficient) were reported, together with the trend line.

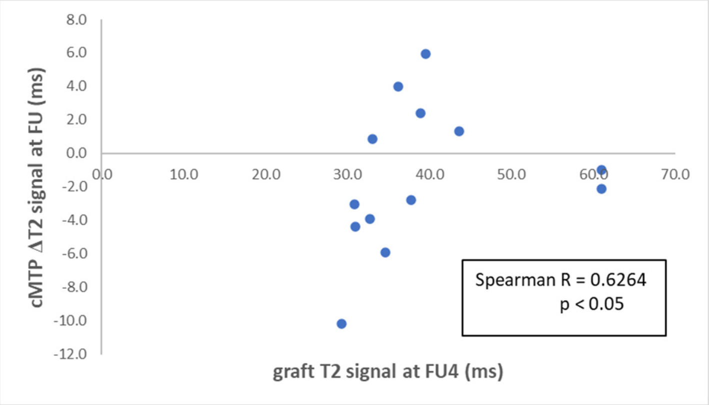

Figure S2. Distribution of the cMTP ΔFU T2 signal (ms) as a function of the graft T2 signal (ms) at FU4. Spearman's correlation details (p-value and R-coefficient) were reported, together with the trend line.

C2. Relationship between cartilage T2 values and knee laxity

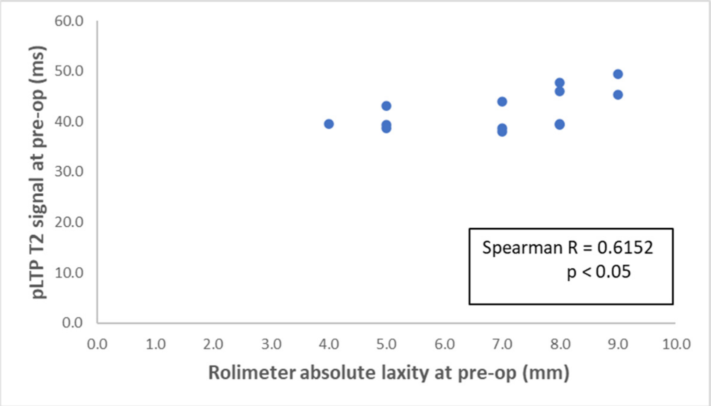

Figure S3. Distribution of the pLTP T2 signal (ms) at pre-op as a function of the absolute laxity (mm) at pre-op, assessed by Rolimeter. Spearman's correlation details (p-value and R-coefficient) were reported, together with the trend line.

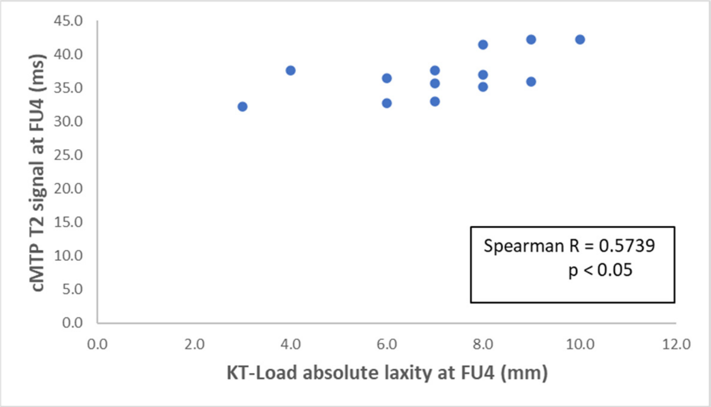

Figure S4. Distribution of the cMTP T2 signal (ms) at FU4 as a function of the absolute laxity (mm) at FU4, assessed by KT-Load. Spearman's correlation details (p-value and R-coefficient) were reported, together with the trend line.

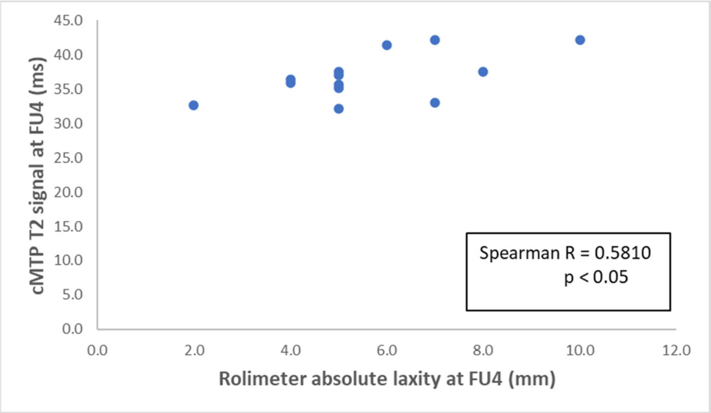

Figure S5. Distribution of the cMTP T2 signal (ms) at FU4 as a function of the absolute laxity (mm) at FU4, assessed by Rolimeter. Spearman's correlation details (p-value and R-coefficient) were reported, together with the trend line.

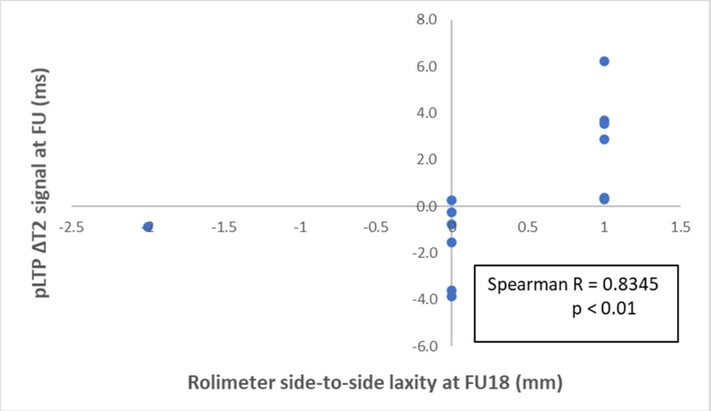

Figure S6. Distribution of the pLTP ΔFU T2 signal (ms) as a function of the side-to-side laxity (mm) at FU18, assessed by Rolimeter. Spearman's correlation details (p-value and R-coefficient) were reported, together with the trend line.

C3. Relationship between graft T2 signal and knee laxity

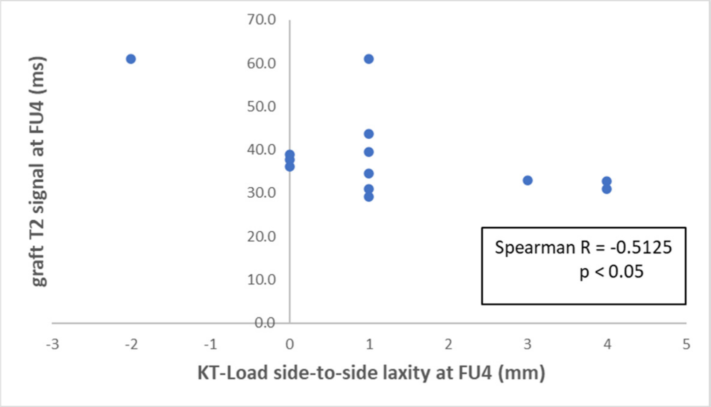

Figure S7. Distribution of the graft T2 signal (ms) at FU4 as a function of the side-to-side laxity (mm) at FU4, assessed by KT-Load. Spearman's correlation details (p-value and R-coefficient) were reported, together with the trend line.

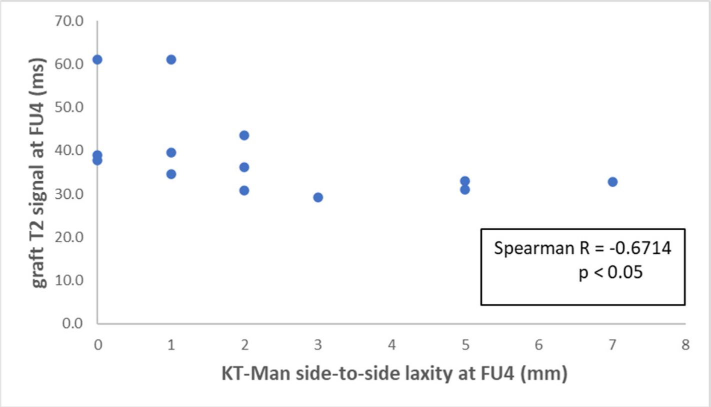

Figure S8. Distribution of the graft T2 signal (ms) at FU4 as a function of the side-to-side laxity (mm) at FU4, assessed by KT-Man. Spearman's correlation details (p-value and R-coefficient) were reported, together with the trend line.

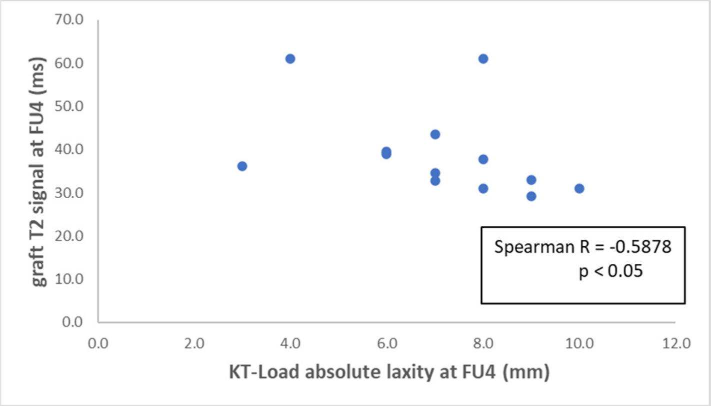

Figure S9. Distribution of the graft T2 signal (ms) at FU4 as a function of the absolute laxity (mm) at FU4, assessed by KT-Load. Spearman's correlation details (p-value and R-coefficient) were reported, together with the trend line.

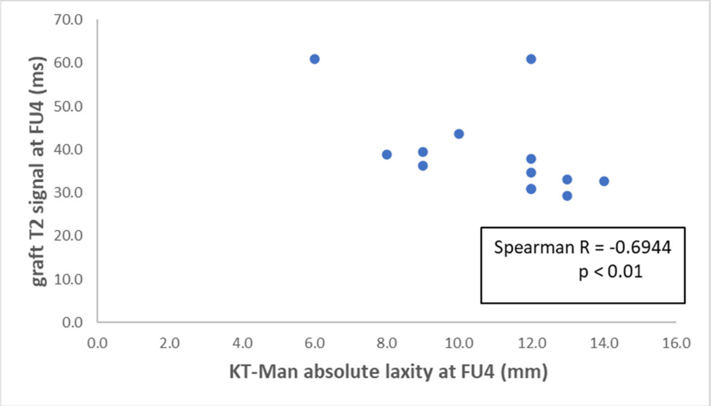

Figure S10. Distribution of the graft T2 signal (ms) at FU4 as a function of the absolute laxity (mm) at FU4, assessed by KT-Man. Spearman's correlation details (p-value and R-coefficient) were reported, together with the trend line.

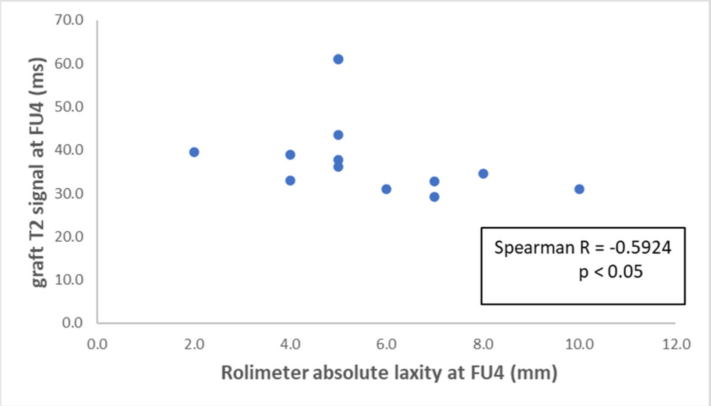

Figure S11. Distribution of the graft T2 signal (ms) at FU4 as a function of the absolute laxity (mm) at FU4, assessed by Rolimeter. Spearman's correlation details (p-value and R-coefficient) were reported, together with the trend line.

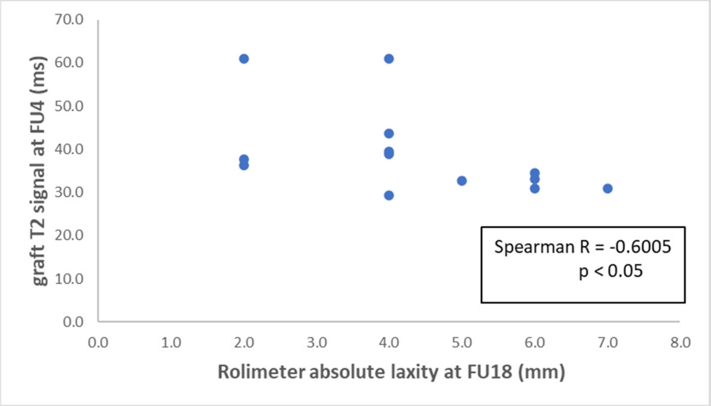

Figure S12. Distribution of the graft T2 signal (ms) at FU4 as a function of the absolute laxity (mm) at FU18, assessed by Rolimeter. Spearman's correlation details (p-value and R-coefficient) were reported, together with the trend line.
